# Research on obstacle avoidance path planning of UAV in complex environments based on improved Bézier curve

**DOI:** 10.1038/s41598-023-43783-7

**Published:** 2023-09-30

**Authors:** Zhihao Zhang, Xiaodong Liu, Boyu Feng

**Affiliations:** 1 Air Traffic Control and Navigation College, Airforce Engineering University, Xi’an, China; 2 Equipment Management and UAV Engineering College, Airforce Engineering University, Xi’an, China

**Keywords:** Electrical and electronic engineering, Applied mathematics

## Abstract

Obstacle avoidance path planning is considered an essential requirement for unmanned aerial vehicle (UAV) to reach its designated mission area and perform its tasks. This study established a motion model and obstacle threat model for UAVs, and defined the cost coefficients for evading and crossing threat areas. To solve the problem of obstacle avoidance path planning with full coverage of threats, the cost coefficients were incorporated into the objective optimization function and solved by a combination of Sequential Quadratic Programming and Nonlinear Programming Solver. The problem of path planning under threat full coverage with no solution was resolved by improving the Bézier curve algorithm. By introducing the dynamic threat velocity obstacle model and calculating the relative and absolute collision cones, a path planning algorithm under multiple dynamic threats was proposed to solve the difficulties of dynamic obstacle prediction and avoidance. Simulation results revealed that the proposed Through-out method was more effective in handling full threat coverage and dynamic threats than traditional path planning methods namely, Detour or Cross Gaps. Our study offers valuable insights into autonomous path planning for UAVs that operate under complex threat conditions. This work is anticipated to contribute to the future development of more advanced and intelligent UAV systems.

## Introduction

In recent years, advancements in technology have made UAVs an essential component of modern-day equipment for human society. The smooth arrival of UAVs at their mission area depends on efficient path planning, which is the core of the flight to the mission area.

Researchers have widely applied Bionic Algorithms to resolve the issue of autonomous path planning for UAVs. Bionic algorithms include particle swarm optimization (PSO)^1–3^, wolf swarm optimization (WSO)^4–7^, and ant colony optimization (ACO)^8^. Although these algorithms exhibit strong robustness and adaptability, they are susceptible to outcomes that are locally optimal^9^. As it facilitates continual directional adjustments^10^, genetic algorithms remain a top choice among many scholars. On the downside, genetic algorithms demand ample internal computational resources and necessitate high computing power^11^. Apart from theoretical studies, scholars have conducted numerous experimental verifications of UAV path planning algorithms across multiple scenarios and tasks^12–15^. Wu’s research focuses on improving the obstacle avoidance capabilities of UAVs through flexible path planning, particularly in disturbed environments^16,17^. Quan and Ji's research accomplished fast adaptive path planning without maps in unknown environments^18,19^. Furthermore, Zhou investigated the architecture of UAV swarms and accomplished their distributed autonomous navigation^20^.

As a result, intelligent algorithms have become prevalent in autonomous path planning of UAVs for obstacle avoidance, leading to significant research breakthroughs. Nevertheless, certain inadequacies still persist in the available literature.

Firstly, many algorithms are prone to falling into local optima. As a result, researchers are striving to improve algorithms and obtain the globally optimal path. However, improved algorithms often lead to increased computation, thereby reducing planning efficiency.

Secondly, previous studies have not found effective solutions to the challenge of achieving full coverage. In existing literature, the setting of obstacles is mostly scattered, and there are gaps between obstacles^17–20^. In fact, when UAVs fly over long distances, some continuous mountain ranges can create obstacles throughout a certain area. When the path is full of threats, the UAV must traverse the threat area with minimal cost. The traditional Detour or Cross Gaps methods are no longer deemed practical.

Thirdly, most studies on path planning only consider the ideal situation of a fixed threat area^13,19^. However, there might be dynamic obstacle areas in real-world situations as well. The straightforward application of the aforementioned algorithms cannot be used to solve path planning issues under dynamic threats.

To resolve the problems mentioned above, this study investigates obstacle avoidance path planning for UAVs in complex conditions. We propose path planning methods for UAVs that encounter both static and dynamic obstacles. The challenge of path planning for UAVs when navigating through dynamic obstacles and full coverage obstructions was addressed using the improved Bézier curve model. Therefore, the proposed method can enhance the ability of UAV path planning and offer a viable approach for UAVs to fly safely even under complex obstacle conditions.

## Modeling

### UAV motion model

This study operated under the assumption that the UAV could be modeled as a rigid body with a constant mass. Furthermore, for the sake of simplicity, the curvature of the Earth was disregarded in the analytical framework. The motion model of the UAV is represented as:

1 $$ \mathop{v}\limits^{\rightharpoonup} _{uav} = (v_{uav} \times \cos \eta ,v_{uav} \times \sin \eta ), $$

2 $$ UAV = (x,y), $$

where $v_{uav}$ is the speed, $\eta$ is the heading angle, and $(x,y)$ denotes the current coordinate position of the UAV.

This study focuses on medium and large fixed-wing UAVs characterized by a high aspect ratio. The parameters governing the UAVs under investigation in this research were formulated in alignment with the performance metrics mentioned earlier, with a speed range of [100–150] $km/h$ and an acceleration range of [− 5, 5]$m/s^{2}$^21–23^.

### Threat area model

This paper focuses on the research of medium and large fixed wing UAVs. The main static obstacles encountered by such UAVs are mountains and tall buildings. In typical scenarios, these obstacles adopt a circular layout. Nevertheless, due to the proximity of certain hindrances, they tend to congregate into clusters, frequently giving rise to non-uniform configurations. The circular threat area, the fan-shaped threat area and coverage angle were set as the radius of $r = 15km$, radius $r = 30km$ and $\theta = 120^\circ$, respectively.

For medium to large UAVs, the dynamic threats are primarily manned and UAVs. In this paper, the dynamic threat area was set as a movable circle. Based on common civil aviation aircraft parameters and safe flight interval, the dynamic obstacles area radius, coverage angle and speed range were set at of $r = 5km$, $\theta = 360^\circ$ and [0, 600]$km/h$.

## Path planning under static full coverage threats

In UAV mission planning, obstacle avoidance is regarded as a top priority. Nevertheless, if crossing a threat area is inevitable, it is critical to cross it while minimizing exposure to the threat. The initial position of UAV was set as $(x_{0} ,y_{0} )$, and the central point of the mission area was set as $(x_{f} ,y_{f} )$. The route to the mission area is punctuated by circular and fan-shaped threat areas. The UAV initiates its journey from the initial position, meticulously fine-tuning control variables while ensuring compliance with constraints. Simultaneously, it strives to fulfill the stipulated objective function, culminating in its successful arrival at the designated mission area.

### Bézier curve model

Enhancing flight performance and mitigating the intricacies of autonomous control necessitate the creation of a streamlined, continuous flight path. In pursuit of this, the second-order Bézier curve has been harnessed as a model for path depiction, entailing the definition of data points and control points^24^. Notably, the efficacy and intricacy of Bézier curve path planning is focused on the count and placement of control points. In light of this, the adoption of the second-order Bézier curve emerges as a judicious choice for path modeling, owing to its elevated precision and rapid computational throughput^25^. The mathematical expression for the second-order Bézier curve is as follows:

3 $$ B(t) = (1 - t)^{2} P_{0} + 2t(1 - t)P_{1} + t^{2} P_{2} ,\,\,\,t \in [0,\,\,1] $$

The data points $P_{0}$, and $P_{2}$ were coordinated, and the control point $P_{1}$, as shown as follows:

4 $$ f(x(t),y(t)) = (1 - t)^{2} (x_{0} ,y_{0} ) + 2t(1 - t)(x_{1} ,y_{1} ) + t^{2} (x_{2} ,y_{2} ),\,\,t \in [0,\,\,1] $$

### Receding planning framework

Bézier curve model represents a localized path planning approach, confining its operation to the trajectory’s initial and terminal points. This tendency yields outcomes of local optimality while neglecting the broader scope of comprehensive planning. In response to this challenge, a backward planning framework was implemented. At each sampling time *t*, an optimized control variable within the finite programming interval $[t,t + T_{p} ]$ was established based on the objective function and input from the previous sampling time. The next control input for interval $[t + 1,t + 1 + T_{p} ]$ was then calculated during the following sampling time *t* + 1. This process was repeated until path planning was complete. The specific planning steps are as follows.

*Step 1* Using the objective function and optimization variables, three path segments within the planning time $[t,t + T_{p} ]$ on the internal Bézier curve were determined in accordance with the current location $[x(t),y(t)]$, and nine data points and control points ($P_{0} ,P_{1} , \cdots ,P_{8}$) were obtained.

*Step 2* The initial path planning within interval $[t,t + T_{e} ]$ was conducted, with determination points $P_{0} ,P_{1} ,$ and $P_{2}$ selected.

*Step 3* At that time, three path segments within time $[t + T_{e} ,t + T_{e} + T_{p} ]$ were re-planned according to the location of UAVs $[x(t + T_{e} ),y(t + T_{e} )]$ on the Bézier curve, and nine data points and control points ($P_{0} ,P_{1} , \cdots ,P_{8}$) were redefined.

*Step 4* Repeat *Step1-Step3* till the UAV reaches the mission area^26^.

### Model optimization and solution

#### Objective function and constraint conditions

When the UAV embarks on a journey through an area blanketed by full threat coverage, the primary objective becomes the minimization of traversal instances while adhering to the following optimization objective function:

5 $$ \min \left[ {\frac{{d_{last - f} }}{\sqrt 2 L} - \frac{\alpha }{{IJI^{\prime}}}\sum\limits_{i}^{m} {\sum\limits_{j}^{1} {\sum\limits_{{i^{\prime} \in D}} 1 } } (d_{{iji^{\prime}}} \le r_{obs(i^{\prime})} ,} \right.\left| {\theta_{{iji^{\prime}}} } \right| \le \theta_{obs(i^{\prime})} )\left. {\frac{{d_{iji^{\prime}} }}{{r_{obs(i^{\prime})} }}} \right], $$

where $d_{last - f}$ represents the distance between the end point and the center point of the current of the mission area, $L$ indicates the side length of the task area, $\sqrt 2 L$ is its diagonal length,* i* is the number of planned Bézier curve segments, $i \in (1,2, \cdots ,m)$, $j$ is the time of each segment, $j \in (0,0.1,0.2, \cdots ,1)$, $i^{\prime}$ is the number of threat areas, $i^{\prime} \in (1,2, \cdots ,D)$. Besides, $d_{iji^{\prime}}$ is the distance between obstacle and UAV, $r_{obs(i^{\prime})}$ denotes the radius of the threat area, $\theta_{iji^{\prime}}$ is the angle between the UAV coordinates and the obstacle coordinates, primarily used to judge the sector threat area. $\theta_{obs(i^{\prime})}$ is the coverage angle of the obstacle $i^{\prime}$. $1(d_{{iji^{\prime}}} \le r_{obs(i^{\prime})} ,\left| {\theta_{{iji^{\prime}}} } \right| \le \theta_{obs(i^{\prime})} )$, determines whether the UAV has entered the threat area by analyzing its position and angle, and assigns a value of 1 in the event that the UAV successfully crosses the threat area. If it does not traverse, the result of this function was defined as 0. Parameter $\alpha$ refers to the cost coefficient, which is used to adjust the weight after normalization.

Facilitating effective UAV path planning mandates a nuanced assessment of an array of constraints, encompassing a spectrum of influential factors such as smoothness, acceleration, obstacles, and speed limitations.

##### Smooth constraint

To facilitate a trajectory marked by fluidity and cohesion, the utilization of Bézier curve subdivision planning is commonplace. This approach mandates the congruence of derivatives between adjoining paths at their junctures, and can be mathematically expressed as follows:

6 $$ B^{\prime}(t = 1)_{i - 1} = B^{\prime}(t = 0)_{i} . $$

##### Acceleration constraint

Constraints must be imposed on the acceleration of UAVs to ensure that their flight performance remains within acceptable parameters. This measure helps to keep the speed changes of UAVs within a reasonable range:

7 $$ a \in [a_{\min } ,a_{\max } ]. $$

##### Speed constraint

During UAV operations, it is crucial that the vehicle can quickly reach its intended mission area while also maneuvering at lower speeds when necessary to avoid threats. In order to achieve this balance and guarantee optimal performance, an appropriate speed range must be determined for the UAV:

8 $$ v \in [v_{\min } ,v_{\max } ]. $$

##### Evade threat area constraints

When a UAV is attempting to evade a threat area, the angle and radius of coverage of any sector or circular hazard must be taken into account. By considering these factors, the UAV can avoid danger to the greatest possible extent. When there are no gaps in the threat area, the UAV should try to move around the edge of the area whenever possible.

9 $$ \left\{ \begin{gathered} d > r_{obs(i^{\prime})} \hfill \\ if \, d \le r_{obs(i^{\prime})} \, and \, \theta_{i} \le \theta_{obs(i^{\prime})} ,d \to r_{obs(i^{\prime})} \hfill \\ \end{gathered} \right. $$

#### Solution

Sequential quadratic programming (SQP) algorithm is commonly used to solve nonlinear programming problems due to its high level of computational efficiency and robust boundary exploration capabilities. This approach can be mathematically expressed as follows:

10 $$ \min f(x)s.t.\left\{ \begin{gathered} g_{u} (X) \le 0,(u = 1,2, \cdots ,p) \hfill \\ h_{v} (X) = 0,(v = 1,2, \cdots ,m) \hfill \\ \end{gathered} \right. $$

The objective function was simplified to a quadratic function at point $X^{k}$ using the Taylor Expansion.

11 $$ \min f(x) = \frac{1}{2}[X - X^{k} ]^{T} \nabla^{2} f(X^{k} )[X - X^{k} ] + \nabla f(X^{k} )^{T} [X - X^{k} ],s.t.\left\{ \begin{gathered} \nabla g_{u} (X^{k} )^{T} [X - X^{k} ] + g_{u} (X^{k} ) \le 0,(u = 1,2, \cdots ,p) \hfill \\ \nabla h_{v} (X^{k} )^{T} [X - X^{k} ] + h_{v} (X^{k} ) = 0,(v = 1,2, \cdots ,m) \hfill \\ \end{gathered} \right. $$

The above objective function requires certain constraints, which can be mathematically expressed as follows:

12 $$ \begin{gathered} \, \min f(x) \hfill \\ s.t.\left\{ \begin{gathered} c(x) \le 0 \hfill \\ ceq(x) = 0 \hfill \\ A \cdot x \le b \hfill \\ Aeq \cdot x = beq \hfill \\ lb \le x \le ub \hfill \\ \end{gathered} \right. \hfill \\ \end{gathered} $$

### Simulations

#### Simulation experiment design

The mission area encompassed a rectangular expanse measuring 100* km* × 100* km*, within which both fan-shaped and circular threat areas were strategically situated. The fan-shaped threat area radius, the coverage angle, circular threat area radius, the coverage angle, the central point coordinate of the mission area and the estimated attack position radius were respectively set at $r_{obs(i^{\prime})} = 30km$, $\theta_{obs(i^{\prime})} = 120^\circ$, $r_{obs(i^{\prime})} = 15km$, $\theta_{obs(i^{\prime})} = 360^\circ$, (100,100) and 10* km*. After UAV enters the mission area, the path planning was completed. Starting from (0,0), the UAV had a speed range of [100,150]$km/h$ and an acceleration range of [− 5, 5]$m/s^{2}$. The simulation step size was $\Delta t = 0.1s$.

**Figure 1:**
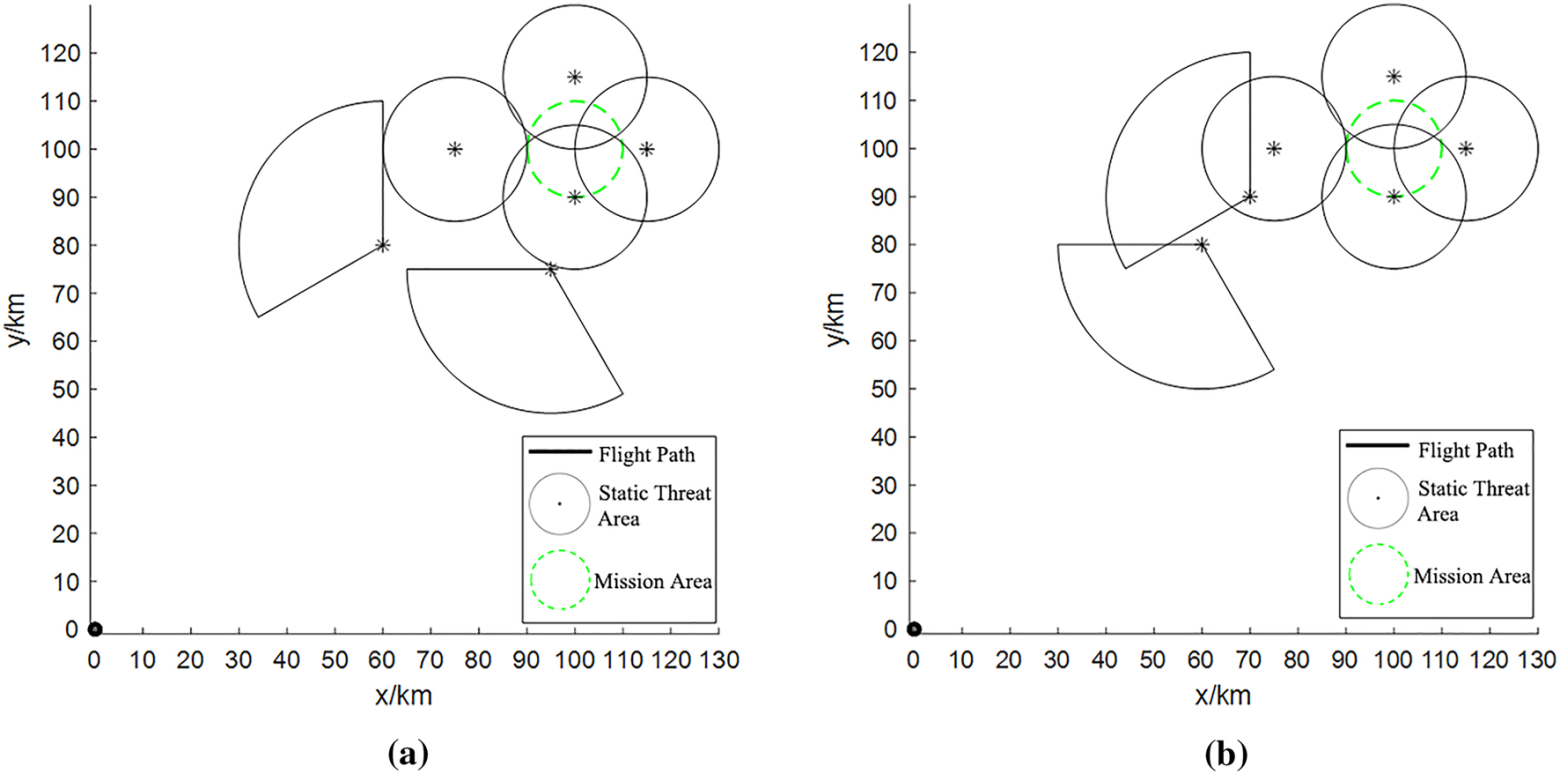
Distribution of threat areas: (**a**) task scenario 1; (**b**) task scenario 2.

In the first task scenario (Fig. 1a), the fan-shaped threat area was defined by the site coordinates (60, 80) and (95, 75), while the circular threat area was defined by the site coordinates (75, 100), (100, 90), (100, 115), and (115, 100). In the second task scenario (Fig. 1b), the site coordinates of the fan-shaped threat area were changed to (60, 80) and (70, 90). It is worth noting, however, that the position of the circular threat area remained unaltered throughout these alterations.

In the context of task scenario 1, the UAV can strategically chart its course through the secure zone nestled between the two fan-shaped threat regions utilizing the “Cross Gaps” method, which serves as a navigation blueprint. However, under task scenario 2, the repositioned fan-shaped threat area obstructs the trajectory of UAVs to the central locus of the mission area. Upon evading this threat area, the UAV’s path necessitates autonomous planning through the aid of the selected algorithm, thereby imposing heightened requisites on its planning prowess.

#### Simulation results and analysis

The simulation experiment was run on a computer that equipped with an eighth-generation i7-6700 K processor, 32 GB RAM, and a 1 TB hard drive. *MATLAB-R2018b* was adopted as the simulation software.

**Figure 2:**
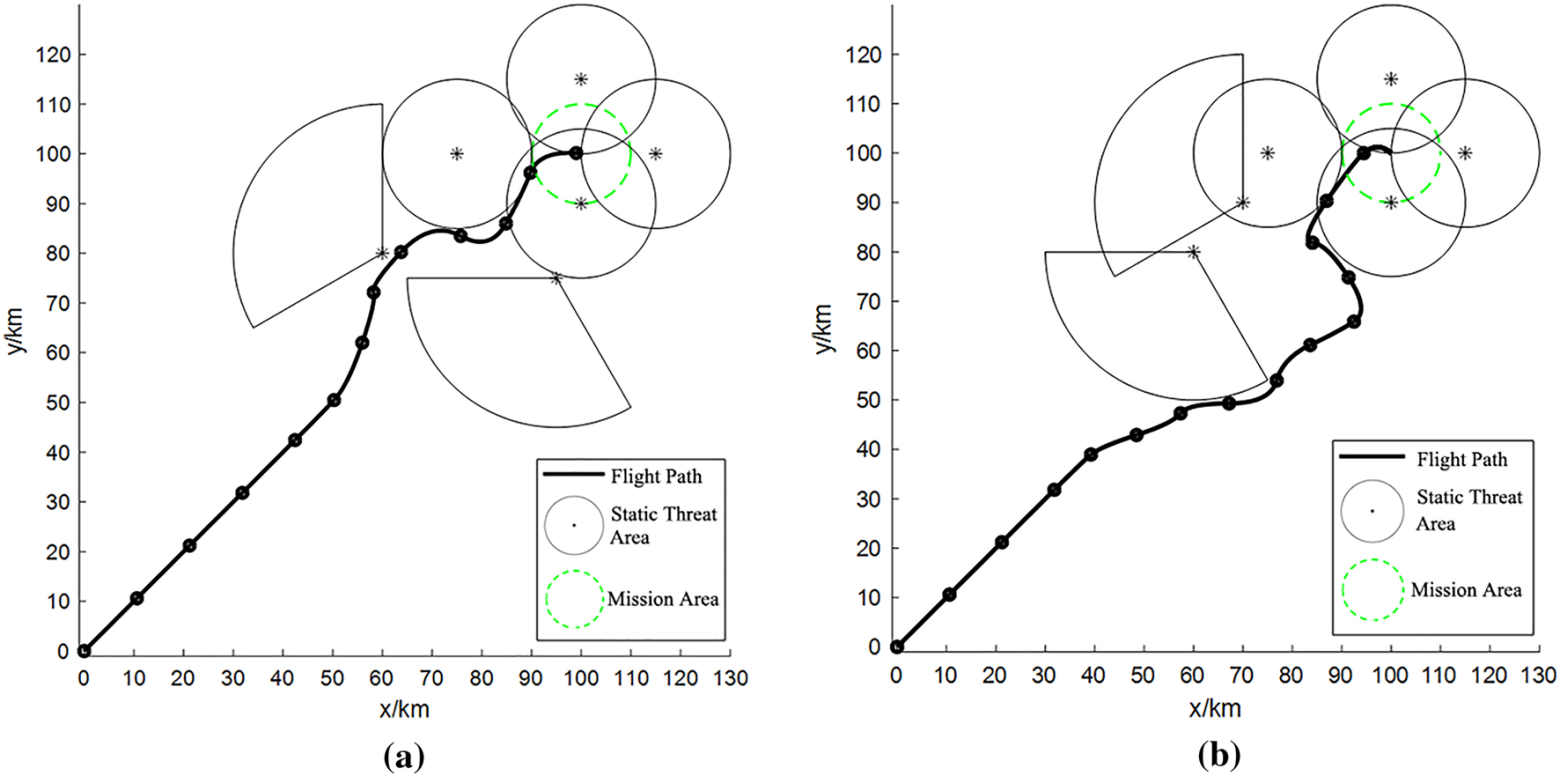
Results of path planning: (**a**) task scenario 1; (**b**) task scenario 2.

Illustrated in Fig. 2a is the outcome of the proposed path planning algorithm for task scenario 1.

The path planning procedure in task scenario 1 was successfully executed in a rapid 5.875 s, yielding a highly favorable outcome. As clearly depicted in Fig. 2a, this plan adeptly circumvents the threat area, achieving comprehensive avoidance. Notably, for the all-encompassing threat area proximate to the central mission locale, the algorithm astutely selects the path with the lowest cost.

Transitioning to task scenario 2, the path planning output generated by the algorithm introduced in this paper is depicted in Fig. 2b. The path planning endeavor for task scenario 2 necessitated a cumulative 8.254 s to yield the optimal plan. While this marked a marginally longer processing time compared to scenario 1, the resultant plan exhibited comparable efficacy. Concretely, the UAV adeptly maneuvers around the fan-shaped threat area, making astute choices among several crossing options while traversing the threat area.

## Path planning under dynamic threats

In addition to full coverage of static threats, UAVs may also encounter dynamic threats brought by other manned or unmanned aerial vehicles during flight, which belong to the complex threat environment faced by UAVs. The path planning of UAV under dynamic threat can also be achieved by improving Bézier curve. Because the position of the dynamic threat changes in real time, it is necessary to establish a speed obstacle model to predict its movement, and then use the improved Bézier curve for segmented path planning.

### Speed obstacle model of dynamic obstacles

**Figure 3:**
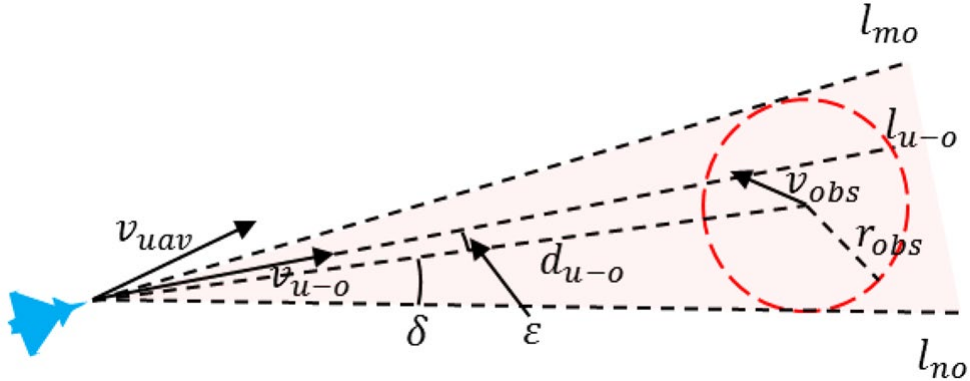
Schematic diagram of speed obstacle model.

Based on the relative motion relationship between the UAVs and the threat area, it can be determined whether the threat can be avoided or not. According to the speed obstacle method^27^, the coordinate position of UAV is $(x_{0} ,y_{0} )$, the central point of the mission area is $(x_{f} ,y_{f} )$, and there is a moving circular threat area in the mission area. Its central position coordinate is $(x_{obs(i^{\prime})} ,y_{obs(i^{\prime})} )$, the radius is $r_{obs(i^{\prime})}$, and the movement speed is $v_{obs(i^{\prime})}$. The speed is $v_{uav}$, the dynamic obstacles area coordinate is $(x_{obs} ,y_{obs} )$, the radius is $r_{obs}$, and the motion speed is $v_{obs}$. Afterwards, the speed obstacle model can be represented, as shown in Fig. 3.

In Fig. 3, $v_{u - o}$ is the relative speed between UAV and dynamic obstacles area,$\mathop{v}\limits^{\rightharpoonup} _{u - o} = \mathop{v}\limits^{\rightharpoonup} _{uav} - \mathop{v}\limits^{\rightharpoonup} _{obs}$. $l_{u - o}$ is a straight line in $v_{u - o}$ direction, $d_{u - o}$ is the distance between the UAV and the center of the threat area, $l_{mo}$ and $l_{no}$ refers to the line between the coordinate position of the UAV and the radius of the threat area. The angle between $d_{u - o}$ and $l_{u - o}$ is $\varepsilon$, and the angle between $d_{u - o}$ and $l_{no}$ is $\delta$. The red area indicates the dynamic obstacles area, which is called relative collision cone (RCC), $RCC = \{ \mathop{v}\limits^{\rightharpoonup} _{u - o} |l_{u - o} \cap r_{obs} \ne \emptyset \}$. When $\mathop{v}\limits^{\rightharpoonup} _{u - o}$ is in the RCC area, i.e., $\varepsilon < \delta$, the UAV needs to avoid the threat area. When $\mathop{v}\limits^{\rightharpoonup} _{u - o}$ is outside the RCC area, that is, $\varepsilon > \delta$, the UAV will refrain from entering the threat area without the changes of both the heading and speed. $\varepsilon ,\delta$ could be calculated by (13) and (14):

13 $$ \delta = \arcsin \frac{{r_{obs} }}{{d_{u - o} }} $$

14 $$ \varepsilon = \arccos \frac{{\mathop{v}\limits^{\rightharpoonup} _{u - o} \cdot \mathop{d}\limits^{\rightharpoonup} _{u - o} }}{{||\mathop{v}\limits^{\rightharpoonup} _{u - o} || \cdot ||\mathop{d}\limits^{\rightharpoonup} _{u - o} ||}} $$

The angle $\delta$ should be smaller than $\pi /2$. When $\delta = \pi /2$, it indicates that the UAV is located at the boundary of the threat area. $\mathop{d}\limits^{\rightharpoonup} _{u - o}$ is perpendicular to the boundary of the relative collision area. When $\delta > \pi /2$, it indicates that the UAV has entered the threat area.

**Figure 4:**
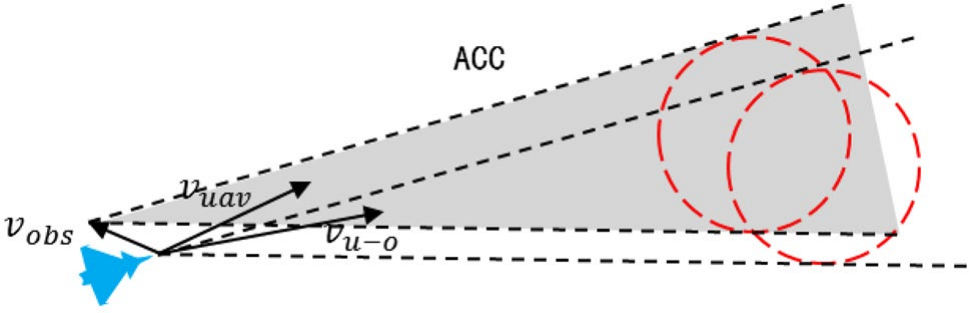
Schematic diagram of absolute collision area.

For the dynamic obstacles area in Fig. 3, after translating $v_{obs}$, the relative collision area RCC can be transformed into absolute collision cone (ACC), as shown in Fig. 4.

Figure 4 illustrates the trajectory of the UAV, depicting its trajectory aligned with the current heading, subsequently leading it into the designated threat area, i.e. $\mathop{v}\limits^{\rightharpoonup} _{uav}$ in ACC area. If the UAV changes its course and speed to make $\mathop{v}\limits^{\rightharpoonup} _{uav}$ outside the ACC area, it can avoid the threat area.

**Figure 5:**
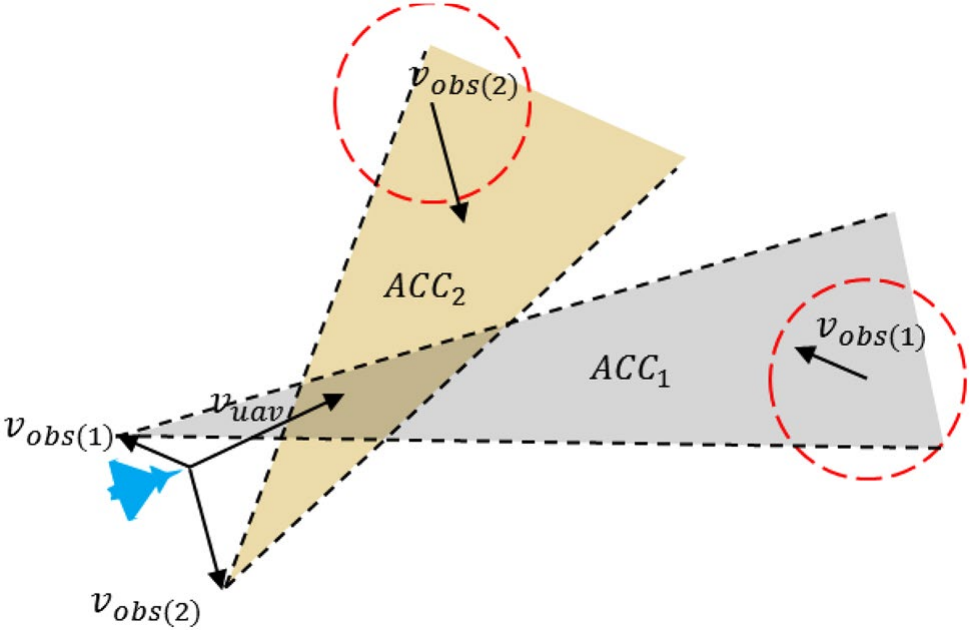
Schematic diagram of multiple absolute collision areas.

In scenarios where the mission area comprises multiple dynamic obstacle areas, it becomes imperative to compute the ACC for varying threat domains, and whether the velocity vector $\mathop{v}\limits^{\rightharpoonup} _{uav}$ of the UAV is within the ACC area was judged. If it is within the area, avoidance is required. Otherwise, there is no need to evade. Multiple absolute collision cone (MACC) was defined as $MACC = \bigcup\nolimits_{i = 1}^{n} {ACC_{i^{\prime}} }$ (Fig. 5).

The crux of the velocity barrier approach in evading threat zones lies in ensuring that the velocity vector $\mathop{v}\limits^{\rightharpoonup}$ of the UAV remains situated outside the realm of absolute collision. This entails strategic adjustments to the UAV's heading and speed. When confronted with multiple dynamic obstacle areas, a concept known as the Multiple Avoidance Collision Cone (MACC) comes into play. To avert potential collisions, the UAV's velocity vector $\mathop{v}\limits^{\rightharpoonup} _{uav}$ must consistently fall outside the boundaries of the MACC region.

The velocity region of UAV can be expressed as:

15 $$ Area_{uav} = \{ \overline{v}_{uav} ,\eta | \, |\overline{v}_{uav} |_{\min } \le |\overline{v}^{\prime}_{uav} | \le |\overline{v}_{uav} |_{\max } ,\,\,\,0 \le \Delta \eta \le \Delta \eta_{\max } \} . $$

### Optimized model

The remaining distance between the UAV and the central nexus of the mission area was employed as the objective function, while the threat level and ACC were deemed the assessment metrics. Guided by predefined parameters encompassing UAV velocity and heading within the threat evasion area, this study embarked on model optimization and computational analysis.

When the UAV evaded the dynamic obstacles area, the following optimization objective function was proposed by combining the evasion strategy and constraints:

16 $$ \min \left[ {\frac{{d_{last - f} }}{\sqrt 2 L} - \frac{1}{{IJI^{\prime}}}\sum\limits_{i}^{m} {\sum\limits_{j}^{1} {\sum\limits_{{i^{\prime} \in D}} {1(\mu (cpa)_{{i^{\prime}}} > 0.6,\mathop{v}\limits^{\rightharpoonup} _{uav(ij)} \cap ACC_{{i^{\prime}}} \ne \emptyset )\left( {\beta \left| {\sin \eta } \right|_{\max } + \gamma \left| {\frac{{v_{uav(j)} - v_{uav(j - 0.1)} }}{{v_{\max } - v_{\min } }}} \right|} \right)} } } } \right]. $$

In (16), the first term indicates that the optimization objective is to minimize the remaining distance between the UAV and the central point of the mission area, and the second term is the objective function to avoid the dynamic obstacles area. Where, $\mu (cpa)_{i^{\prime}}$ is the assessed risk of the $i^{\prime}$ th dynamic obstacles area, $\mathop{v}\limits^{\rightharpoonup} _{uav}$ is the current velocity vector of the UAV, and $ACC_{i^{\prime}}$ represents the absolute collision area. Upon fulfillment of the evasion criteria, the UAV adeptly circumvented the designated threat area. The parameter $|\sin \eta |_{\max }$ indicates that the UAV adjusts the angle to avoid the maximum heading angle in the dynamic obstacles area. $v_{uav(j)} - v_{uav(j - 0.1)}$ represents the speed change of UAV during evasion. The parameters $\beta$ and $\gamma$ are cost coefficients. The weight of course angle and speed changes were adjusted, summed them with $\sum\nolimits_{i}^{m} {\sum\nolimits_{j}^{1} {\sum\nolimits_{i^{\prime} \in D} {} } }$, and then normalized them with $1/IJI^{\prime}$.

### Simulations

#### Simulation experiment design

The task area was as $100km \times 100km$ and 3 circular dynamic obstacles areas were set up within the task area. Threat area radius is $r_{obs(i^{\prime})} = 5km$, coverage angle is $\theta_{obs(i^{\prime})} = 360^\circ$, velocity interval is [0,50]$km/h$, acceleration interval is $[ - 1,1]m/s^{2}$, and angular velocity interval is[− 0.2,0.2]$rad/s$. The UAV initiated its journey from the origin point (0,0), with its speed range spanning from 100 to 150 $km/h$, the acceleration interval was [− 5,5]$m/s^{2}$, the detection distance was $45km$, and the simulation step size was $\Delta t = 0.1s$.

**Figure 6:**
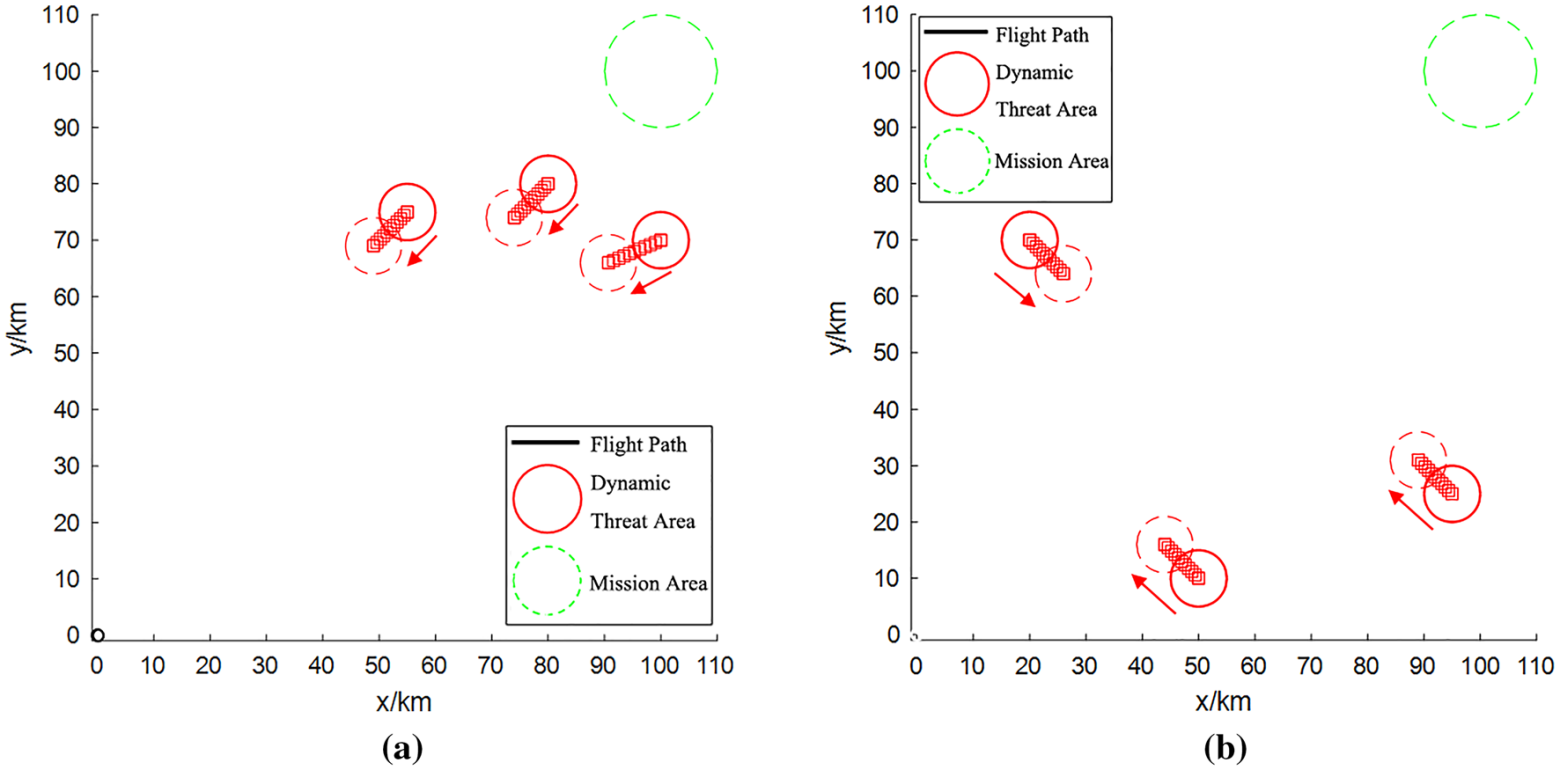
Distribution of dynamic threat areas: (**a**) task scenario 1; (**b**) task scenario 2.

In Fig. 6, the current position of the dynamic obstacle area is visually represented by the red solid circle, while the subsequent position of the threat area is indicated by the red dashed circle.In task scenario 1, the coordinates of the dynamic obstacles area are (55, 75), (80, 80), (100, 70), and its velocity vector is (– 6, − 6), (− 6, − 6), (− 9, − 4). In task scenario 2, the coordinates of the dynamic obstacles area are (20, 70), (50, 10), (95, 25), and its velocity vector is (6, − 6), (− 6, 6), (− 6, 6). In task scenario 1, a distinct configuration was implemented. Furthermore, in task scenario 2, the dynamic obstacle area exhibited a lateral movement relative to the UAV. Consequently, the task of devising a path to circumvent these threats becomes notably more intricate.

#### Simulation results and analysis

**Figure 7:**
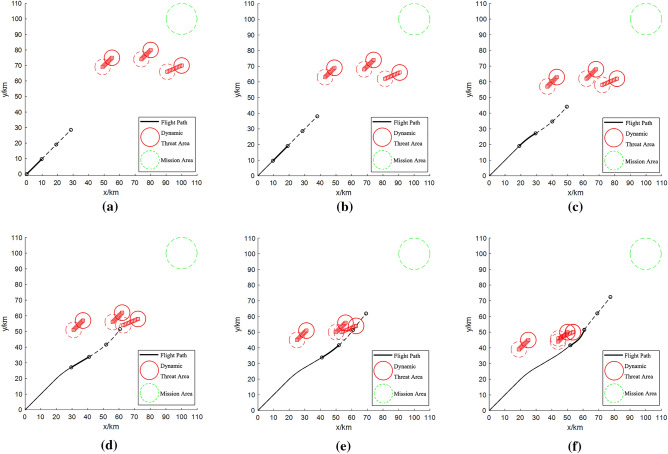
Results of path planning for task scenario 1: (**a**) path segment 1; (**b**) path segment 2; (**c**) path segment 3; (**d**) path segment 4; (**e**) path segment 5; (**f**) path segment 6.

**Figure 8:**
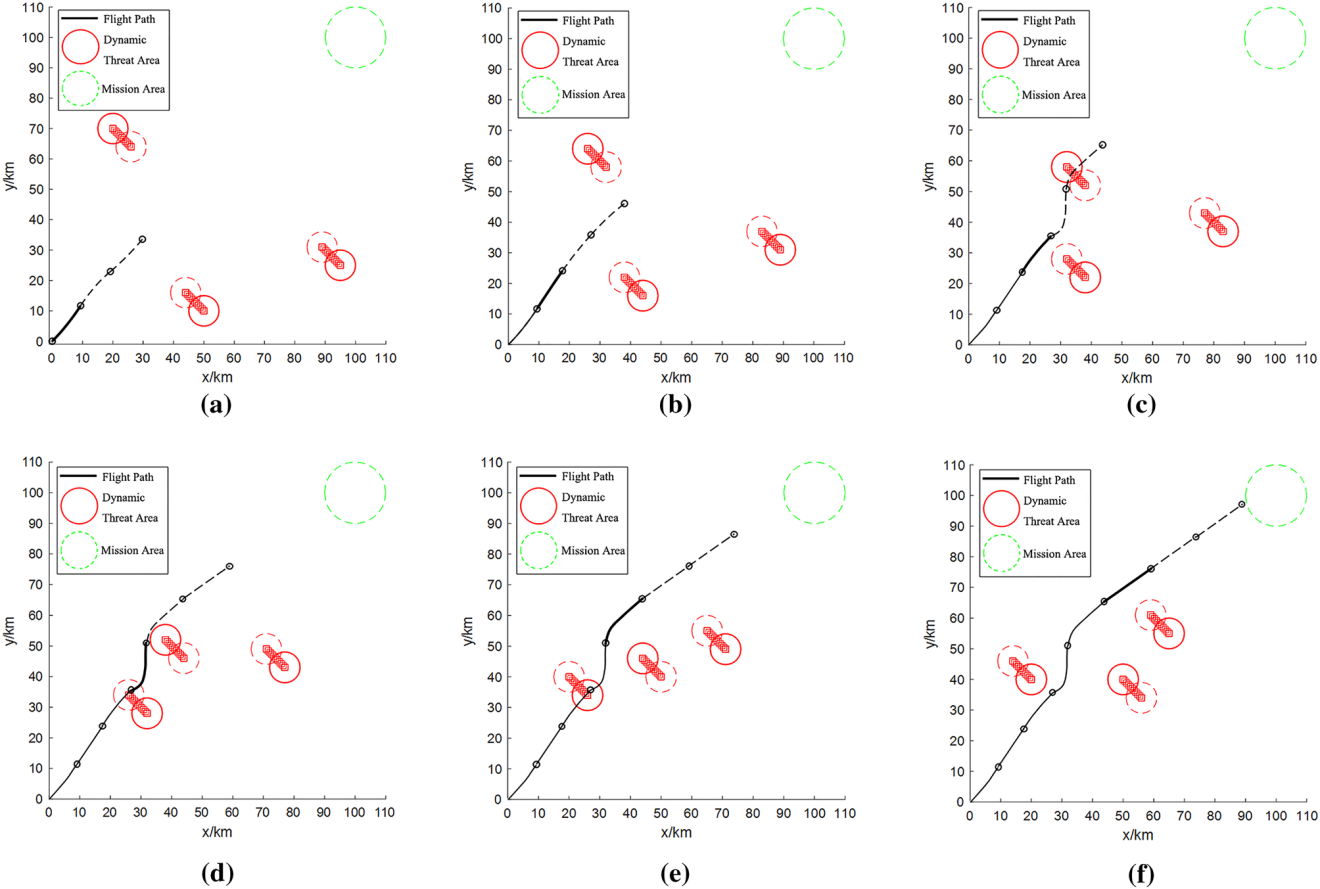
Results of path planning for task scenario 2: (**a**) path segment 1; (**b**) path segment 2; (**c**) path segment 3; (**d**) path segment 4; (**e**) path segment 5; (**f**) path segment 6.

For task scenario 1, the path of algorithm planning in this chapter is shown in Fig. 7:

From Fig. 7, the contiguous flight path segment of the UAV is depicted by the solid black line. Significantly, the bold solid black line demarcates the ongoing path segment of the UAV. In parallel, the dashed black line signifies the meticulously mapped but as yet untraversed path segment. Within Fig. 7d and e, guided by the movement trajectory of the threat area, the UAV’s forthcoming planned path navigates through the prevailing threat area. In the scenario depicted in Fig. 7f, the UAV executed the previously outlined path planning strategy, successfully circumventing potential threats. The threat is moving, and the UAV needs to determine the direction of the threat at any time, so we adopt the method of subsection planning. It takes 7.33 s to complete the six section planning shown in Fig. 7. For task scenario 2, the path planned by the algorithm in this paper is shown in Fig. 8.

In the context of task scenario 2, the motion logic governing dynamic obstacle area mirrors that of task scenario 1. To circumvent the dynamic obstacle areas on both the left and right flanks, parameters $\beta = 5$ and $\gamma = 10$ were defined. As depicted in Fig. 8d, adjustments were notably made to flight speed and heading angle in alignment with the path planning objective function and threat avoidance cost function. This orchestrated adaptation enabled the UAV to adeptly sidestep the dynamic obstacles. It takes 9.56 s to complete the six section planning shown in Fig. 8.

Drawing insights from Figs. 7 and 8, it becomes apparent that the UAV can successfully predict the threat direction and implement obstacle avoidance path planning when dealing with two kinds of dynamic threats in the same and different directions. When the number of dynamic threats is less than 3, the time to complete the obstacle avoidance planning is less than 10 s. This confirms the effectiveness of the path planning algorithm proposed in this article against dynamic threats.

## Conclusions and outlook

The Throughout path planning model was proposed to solve the problem that the traditional Cross Gaps method cannot fulfill the path planning requirements in the presence of complex obstacles faced by the UAV. The path planning of UAV in static threat area is analyzed, and the overall framework of UAV path planning is established. Based on Bézier curve and receding planning framework, combined with the actual situation of UAV obstacle avoidance flight, an optimization model is proposed and constraints are set. By setting the optimization variables and cost functions in the optimization model, the UAV can break through the threat of full coverage. In order to solve the optimization model, the sequential quadratic programming method and the nonlinear programming solver Fmincon are used for calculation. Experiments show that the proposed method can select the most reasonable path in seconds and complete the path planning.

Additionally, the problem of path planning for a UAV facing dynamic obstacles was investigated. The cost function for the velocity and heading angle of the UAV was developed to avoid areas with dynamic obstacles, and a new path planning algorithm was introduced. Simulation results show that the algorithm can reasonably predict the threat movement trend and plan a reasonable escape path for the two kinds of dynamic threats with the same direction and different directions.

The research needs to be improved on two aspects. Firstly, the threat area has been reduced to a two-dimensional plane area, and a three-dimensional area should be created to give more detailed direction on path planning. Secondly, static and dynamic obstacles can be combined and arranged in the same scene for more realistic path planning simulations, especially under complex conditions.

## Data Availability

The main data used in this article is listed in the article. Readers should contact the correspondent if detailed data is required.
